# Risk assessment of hyperbilirubinemia using a three-factor model after cardiac surgery

**DOI:** 10.1186/s12893-024-02731-6

**Published:** 2025-02-13

**Authors:** Weibin Lin, Bin Chen, Kevin Yang, Liting Kuang

**Affiliations:** 1 Department of Cardiac Surgery, The First Affiliated Hospital, Sun Yat-sen University, Guangzhou, Guangdong China; 2 Department of Liver Surgery, The First Affiliated Hospital of Sun Yat-Sen University, Guangzhou, Guangdong China; 3 Zhongshan School of Medicine, Sun Yat-sen University, Guangzhou, Guangdong China; 4 Department of Anesthesiology, The First Affiliated Hospital, Sun Yat-sen University, Guangzhou, Guangdong China

**Keywords:** Internal validation, Blood transfusion, Total bilirubin, Cardiac surgery

## Abstract

**Background:**

Hyperbilirubinemia (HB) is a common occurrence after cardiopulmonary bypass and often results in increased rates of complications.

**Methods:**

We conducted a study on 411 patients who underwent cardiac surgery with extracorporeal circulation and divided them into a training set and a validation set. Least absolute shrinkage and selection operator (LASSO) regression was employed to screen out variables. Multivariate logistic regression was subsequently applied to establish prediction models, which were then evaluated using receiver operating characteristic (ROC) curves and calibration plots. Finally, restricted cubic splines (RCSs) curve pairs were used to calculate the adjusted ORs for each independent risk factor.

**Results:**

A clinical prediction model based on preoperative bilirubin elevation, aortic clamp time and intraoperative red blood cell (RBC) transfusion was successfully developed. The model presented an ROC of 0.855 (0.808, 0.901) in the training set. The validation set demonstrated a promising ROC of 0.839 (0.761, 0.917), and the calibration curve closely approximated the true incidence. The risk of postoperative HB was increased when the aortic clamp time was greater than 133 min, when the red blood transfusion volume was greater than 275 ml or when the preoperative total bilirubin concentration was greater than 16.65 mol/L.

**Conclusion:**

In this study, the aortic clamp time was identified as a crucial factor for postoperative HB during cardiac surgery. A three-factor prediction model that includes the aortic clamp time for the prediction of HB after cardiac surgery was successfully established. Furthermore, patients with hyperbilirubinemia post-surgery had a worse short-term prognosis.

**Supplementary Information:**

The online version contains supplementary material available at 10.1186/s12893-024-02731-6.

## Background

Hyperbilirubinemia (HB) is a common occurrence after extracorporeal circulation, typically manifesting within 1 week after surgery. It was defined as a threshold of total bilirubin greater than 3 mg/dl within 7 days after extracorporeal circulation surgery. The incidence of postoperative HB ranges from 10.1 to 35.1%. This complication often results in increased rates of renal insufficiency, pulmonary infections, and in-hospital mortality [1–5]. Despite further improvements in surgical techniques, postoperative HB after extracorporeal circulation still occurs frequently [6]. Many studies have identified risk factors for postoperative HB, including preoperative hepatic insufficiency, prolonged circulation time, number of valves replaced, increased right atrial pressure, volume of blood transfusion, advanced age, diabetes mellitus, extracorporeal circulation time and aortic clamp time [5, 7]. However, a clinical prediction model of hyperbilirubinemia (HB) after extracorporeal circulation surgery is not available. Therefore, establishing a model for hyperbilirubinemia (HB) after extracorporeal circulation surgery is imperative.

## Methods

### Study population and demographic characteristics

**Fig. 1:**
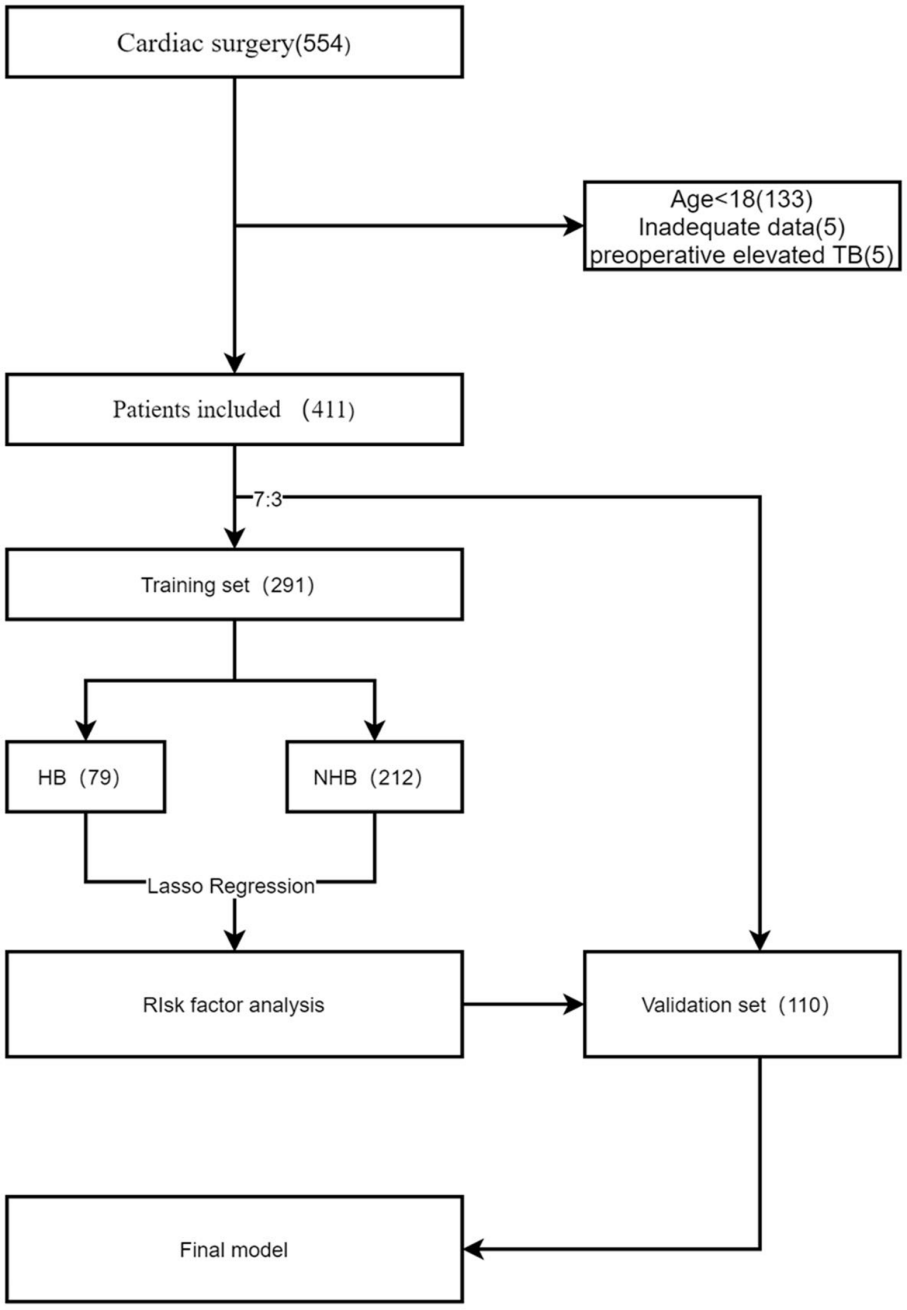
Flow chart of the study

Data from surgical patients in the Department of Cardiac Surgery of the First Affiliated Hospital of Sun Yat-sen University were retrospectively collected from January 2021 to December 2021. This was a retrospective observational cohort study using deidentified data; therefore, the IRB did not require consent from the patients. The inclusion criteria were as follows: aged greater than 18 years and underwent direct cardiac surgery assisted by extracorporeal circulation. The exclusion criteria included patients whose preoperative total bilirubin level was greater than 3 mg/dl and who were missing postoperative total bilirubin data. According to the C statistic of 0.8, 8 possible risk factors were expected to be included, and the estimated sample size was 303 on the basis of the HB incidence of 0.253 from previous literature [8]. This study was approved by the ethics committee of the First Affiliated Hospital of Sun Yat-sen University (No. [2023]493). The data were completely anonymised to remove any identifying information, exempting the informed consent form. The study complied with all regulations and complied with the Helsinki Declaration. The flow chart of the study is shown in Fig. 1.

Open heart surgeries at our center were performed primarily through a median incision to establish extracorporeal circulation. Normothermia or mild hypothermia under an aortic clamp is necessary for myocardial protection, which is conducted mainly by intermittent downstream perfusion of blood cardioplegia or histidine-tryptophan-ketoglutarate (HTK) fluid. Depending on the duration of the aortic clamp, the total reperfusion time was 1/3 of the ACC time. Once the aortic block clamp has been opened, the anesthesiologist begins to administer positive inotropic/vasopressor medications. If a normal cardioversion time is reached, the extracorporeal circulation is tapered down to support flow and withdrawn, and mechanical circulatory support (IABP and ECMO) is initiated when there is complex CPB separation [9]. Postoperatively, patients are usually transferred to a care unit and later to the general ward after tracheal extubation.

Demographic parameters, comorbidities, and laboratory tests were retrieved from the electronic medical records system. Postoperative HB was defined with reference to previous studies with a threshold of total bilirubin greater than 3 mg/dl within 7 days after extracorporeal circulation surgery [8]. The criteria for the definition of AKI refer to our previous literature [10].

### Statistical analysis

There were only 13 missing data points (< 5%) in our data (LV systole 9/411; RV Relax 4/411), which were supplemented with missing values via multiple interpolation before further statistical analysis. All the statistical analyses were performed using R software version 4.2.2. The sample size was calculated using the methodology of previous studies [11]. The normal distribution of the data was assessed using the Kolmogorov–Smirnov test. Continuous variables were reported as the mean ± standard deviation (SD) and were compared via Student’s independent t test. Nonnormally distributed data are reported as the median [interquartile range (IQR)] and were tested by a nonparametric test (Mann‒Whitney *U* test). Categorical variables are presented as numbers and percentages [n (%)] and were compared using the chi-square test or Fisher’s exact test.

All datasets were randomly divided into training and validation cohorts at a 7:3 ratio, and homogeneity analyses were performed between the two cohorts. A sample size of at least ten times the number of characteristics considered in the multivariate analysis is often necessary. As shown in the findings section, we identified 17 characteristics that varied for inclusion in the multifactorial analysis after univariate analysis (all preoperative and intraoperative variables). Therefore, according to the above principle, we must have at least 170 positive cases, whereas the training set had only 79 cases. In this case, the “rule” of “ten” events per variable cannot apply. In other words, we cannot incorporate as many variables into a multifactor regression. To reduce the number of candidate predictors and select the final variables for multivariate logistic regression, the least absolute shrinkage and selection operator (LASSO) method was used [12, 13]. The penalty term was determined by 10-fold cross-validation, selecting the penalty that yielded the smallest mean square error (“glmnet” package). The main tuning parameter was set as follows: type. Measure (loss to use for cross-validation) = “default”, family=“binomial”, nfolds = 10. The optimal model, with the fewest variables, was identified on the basis of λ1 se as the criterion. These variables were subjected to univariate analysis to calculate the OR and *P* values. Factors with *P* < 0.05 in the univariate analysis were included in the subsequent multivariate analysis. The variables that were significant in the multivariate logistic regression model were recognized as variables associated with HB and were used in the final model.

A nomogram was also established with the final model through the “rms” package of R software. The predicted probabilities from the final models were further analyzed using ROC analysis, and the area under the curve (AUC), sensitivity, specificity, positive predictive value (PPV), negative predictive value (NPV) and Youden’s index were reported. Calibration plots (1000 bootstrap resamples) and the Hosmer‒Lemeshow goodness-of-fit test (HL test) were used to evaluate the accuracy of the prediction model in the training cohort and validation cohort. A *P value* > 0.05 indicated that the model calibration degree was reliable. Decision curve analysis (DCA) was performed to assess the clinical usefulness of the predictive model. Restricted cubic splines (RCSs, “plotrcs” package) were used to examine the nonlinear associations and reference points between risk factors and HBs. Furthermore, *P* < 0.05 was considered statistically significant.

## Results

### Overview of the overall situation

A total of 411 patients were enrolled in our study, with 291 and 120 patients assigned to the training and validation cohorts, respectively. The overall incidence of postoperative HB was 28.2% (116/411).

**Fig. 2:**
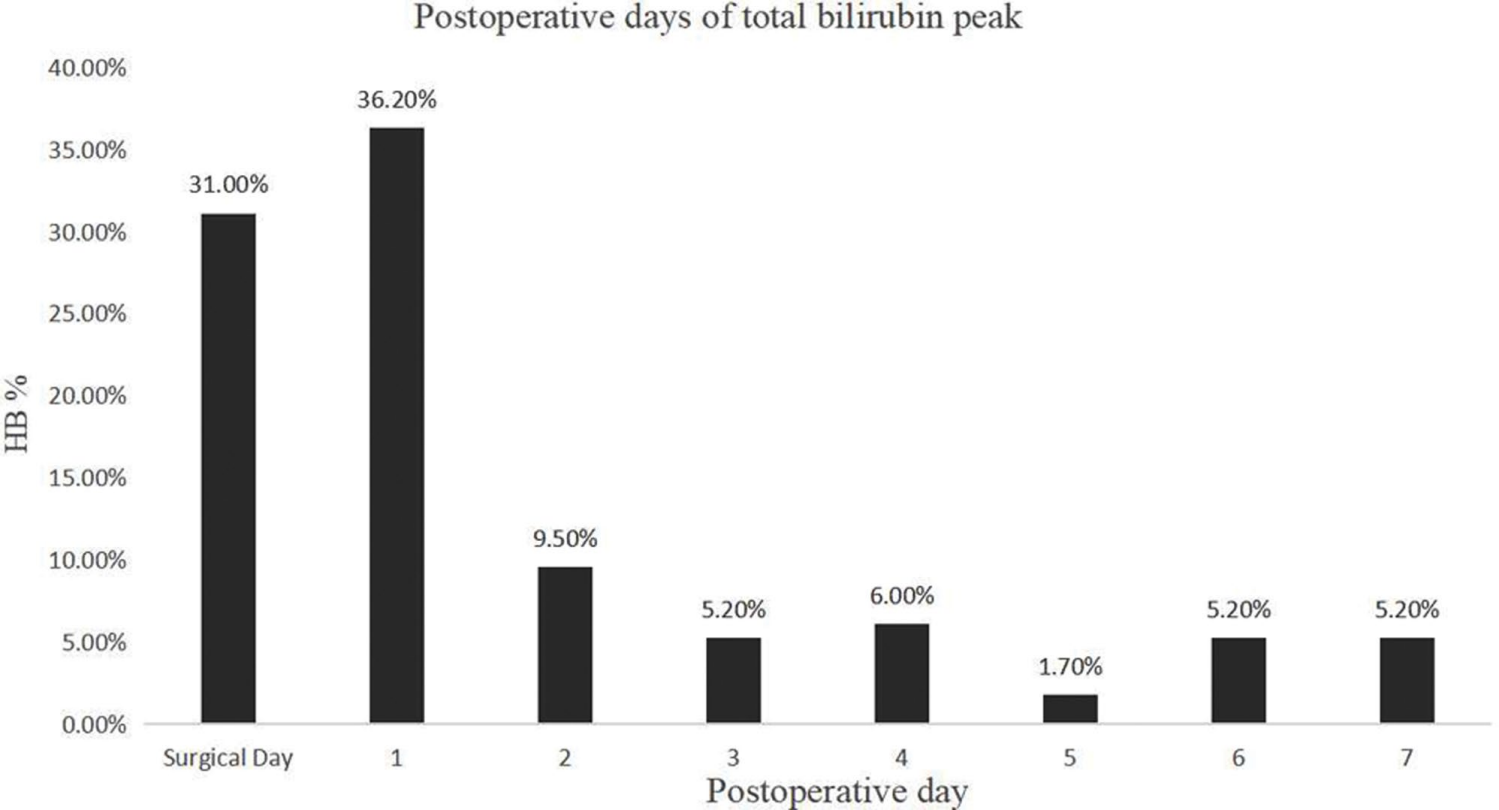
Profile of the high bilirubin peak from the day of surgery to the 7th day after surgery

The peak postoperative total bilirubin level was observed on the day after surgery and the first day after surgery, with incidence rates of 31.0% and 36.2%, respectively (*P* < 0.001) (Fig. 2).

**Fig. 3:**
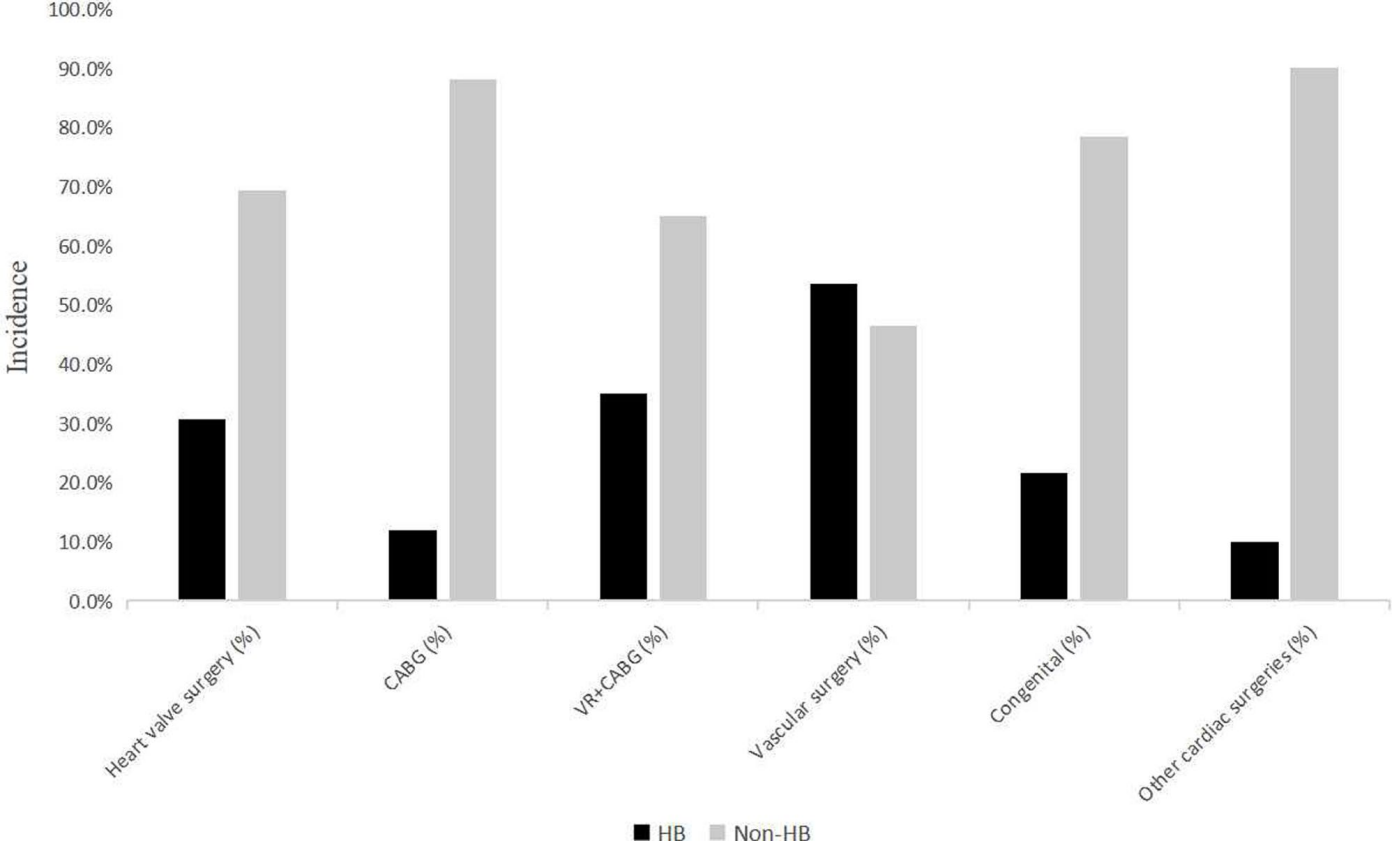
Incidence of postoperative HB in different cardiac surgeries. HB: black bar. Non-HB: gray bar

The incidence of postoperative HB varied among different cardiac surgeries (Table S1). The postoperative HB rates of heart valve surgery, coronary artery bypass grafting (CABG), VR plus CABG, vascular surgery, congenital surgery, and other cardiac surgeries (tumor, heart transplant, etc.) were 30.7%, 11.9%, 35.0%, 53.5%, 21.6%, and 10.0%, respectively (Fig. 3). CABG surgery was associated with lower postoperative HB (*P* < 0.001), whereas vascular surgery seemed to be more prone to postoperative HB (*P* < 0.001). Compared with heart valve surgery, CABG, congenital surgery, and vascular surgery were associated with a greater incidence of postoperative HB (all *P* < 0.05).

### Patient demographic and clinical characteristics

Compared with those in the validation cohort, patients in the training cohort had a better model for the prognosis of end-stage liver disease (MELD) (*P* < 0.05). However, between the two cohorts, there was no significant difference in other preoperative indicators (*P* > 0.05). Furthermore, patient demographic and clinical characteristics showed good homogeneity between the training and validation cohorts (Table 1). There was no significant difference in surgery time, cardiopulmonary bypass (CPB) time, aortic occlusion time, minimum temperature, sevoflurane inhalation, dexmedetomidine use, dopamine use, dobutamine use, norepinephrine use, adrenaline use, various cardiac surgeries, blood loss, transfusions of red blood cells (RBCs), plasma, cryoprecipitates or platelets (PLTs) between the two cohorts (*P* > 0.05). The intraoperative results were also homogenous between the two cohorts (Table 2).

**Table 1:** Patient’s demographic and clinical characteristics in training cohort and validation cohort

Parameters	T	Training cohort		*P*	Validation cohort		*P*	*P* (T: V)
	All (*n* = 411)	Non-HB (*n* = 212)	HB (*n* = 79)		Non-HB (*n* = 83)	HB (*n* = 37)		
Gender, (%)				0.758			0.816	0.779
Male	256 (62.3)	130 (61.3)	50 (63.3)		52 (62.7)	24 (64.9)		
Female	155 (37.7)	82 (38.7)	29 (36.7)		31 (37.3)	13 (35.1)		
Age, y	53.31 ± 13.31	53.35 ± 13.83	54.42 ± 10.48	0.534	52.47 ± 14.86	52.62 ± 12.34	0.957	0.438
BMI, Kg/m^2^	23.09 ± 3.58	23.04 ± 3.38	23.25 ± 3.62	0.645	23.51 ± 4.13	22.03 ± 3.23	0.056	0.895
ASA grade, (%)				0.043			0.775	0.263
2	8 (1.9)	6 (2.8)	1 (1.3)		1 (1.2)	0 (0.0)		
3	303 (73.7)	167 (78.8)	53 (67.1)		58 (69.9)	25 (67.6)		
4	98 (23.8)	39 (18.4)	24 (30.4)		23 (27.7)	12 (32.4)		
5	2 (0.5)	0 (0.0)	1 (1.3)		1 (1.2)	0 (0.0)		
MELD score	18.03 ± 3.12	17.21 ± 2.80	19.42 ± 2.87	< 0.001	17.46 ± 2.46	21.10 ± 3.85	< 0.001	0.022
ALBI score	-2.52 ± 0.36	-2.57 ± 0.34	-2.45 ± 0.31	0.006	-2.54 ± 0.40	-2.37 ± 0.39	0.028	0.198
APRI score	0.40 ± 0.59	0.35 ± 0.41	0.43 ± 0.43	0.182	0.39 ± 0.61	0.66 ± 1.27	0.119	0.123
FIB-4 score	1.61 ± 1.44	1.47 ± 1.02	1.84 ± 1.25	0.009	1.64 ± 2.24	1.85 ± 1.52	0.601	0.377
EF, %	66.63 ± 10.90	64.01 ± 10.91	63.14 ± 11.52	0.552	63.29 ± 10.49	63.30 ± 10.79	0.997	0.684
Smoke (%)	100 (24.3)	57 (26.9)	15 (19.0)	0.165	16 (19.3)	12 (32.4)	0.116	0.762
Disease								
Atrial.fibrillation (%)	68 (16.5)	32 (15.1)	18 (22.8)	0.122	8 (9.6)	10 (27.0)	0.014	0.588
PVD (%)	31 (7.5)	18 (8.5)	0 (0.0)	0.007	11 (13.3)	2 (5.4)	0.201	0.105
PH (%)	141 (34.3)	67 (31.6)	28 (35.4)	0.535	31 (37.3)	15 (40.5)	0.740	0.269
PCI (%)	45 (10.9)	29 (13.7)	8 (10.1)	0.418	6 (7.2)	2 (5.4)	0.712	0.074
RFD (%)	44 (10.7)	22 (10.4)	8 (10.1)	0.950	8 (9.6)	6 (16.2)	0.300	0.686
Emergency (%)	11 (2.7)	3 (1.4)	5 (6.3)	0.023	2 (2.4)	1 (2.7)	0.924	0.887
AHF (%)	11 (2.7)	6 (2.8)	1 (1.3)	0.439	3 (3.6)	1 (2.7)	0.797	0.596
Preoperative vasoactive (%)	53 (12.9)	23 (10.8)	9 (11.4)	0.895	13 (15.7)	8 (21.6)	0.428	0.074
Diabetes (%)	49 (11.9)	32 (15.1)	6 (7.6)	0.091	6 (7.2)	5 (13.5)	0.271	0.268
Laboratory test								
Hb, g/L	131.90 ± 20.99	132.50 ± 19.38	132.49 ± 22.30	1.000	131.04 ± 24.44	129.19 ± 23.52	0.700	0.374
PLT, ×10^9^/L	219.71 ± 69.34	220.52 ± 65.60	210.01 ± 68.65	0.231	226.05 ± 72.74	221.57 ± 83.50	0.767	0.353
Creatinine, µmol/L	91.58 ± 66.58	92.59 ± 79.39	87.32 ± 32.67	0.568	86.61 ± 39.41	101.59 ± 86.68	0.260	0.840
Albumin, g/L	38.69 ± 4.12	38.74 ± 3.85	38.82 ± 3.72	0.884	38.71 ± 4.90	38.02 ± 4.57	0.465	0.551
TBIL, µmol/L	16.01 ± 7.97	13.41 ± 5.79	21.38 ± 9.64	< 0.001	18.17 ± 9.74	18.93 ± 9.65	0.614	0.840
ALT, U/L	21.0(15.0,32.0)	21.0(15.0,30.0)	21.0(16.0,34.5)	0.355	22.0(16.0,33.0)	23.0(17.0,33.0)	0.621	0.117
AST, U/L	24.0(19.0,31.0)	22.0(18.0,28.0)	27.0(21.5,33.0)	< 0.001	24.0(19.0,31.0)	28.0(21.0,37.0)	0.023	0.225
UA, µmol/L	419.58 ± 137.74	411.01 ± 130.65	436.71 ± 121.74	0.130	417.72 ± 145.69	436.22 ± 184.92	0.557	0.716
INR	1.12 ± 0.28	1.09 ± 0.29	1.14 ± 0.24	0.198	1.07 ± 0.18	1.33 ± 0.44	< 0.001	0.129
NT-proBNP	374.1(99.4,1351.5)	258.5(94.7,1064.0)	580.1(145.2,1797.0)	0.018	376.7(64.2,1216.0)	903.4(205.0,1799.0)	0.045	0.823
HB, %	116(28.2)	—	79 (27.1)	—	—	37 (30.8)		0.450

*Abbreviations* AHF, acute heart failure; ALBI, albumin-bilirubin; ALT, alanine amiotransferase; APRI, AST to Platelet Ratio Index; AST, aspartate transaminase; BMI, Body mass index; BUN, blood urea nitrogen; EF, ejection fraction; FIB-4, fibrosis4; HB, hyperbilirubinemia; Hb, Hemoglobin; INR, International normalized ratio; MELD, Model for end-stage liver disease; NT-proBNP, N-terminal brain natriuretic peptide; PCI, percutaneous coronary intervention; PH, pulmonary hypertension; PLT, platelet; PVD, peripheral vascular disease; RFD, renal function damage; TBIL, total bilirubin; UA, uric acid

**Table 2:** Patient’s intra-operative results in training cohort and validation cohort

Parameters		Training cohort		*P*	Validation cohort		*P*	*P* (T: V)
	All (*n* = 411)	Non-HB (*n* = 212)	HB (*n* = 79)		Non-HB (*n* = 83)	HB (*n* = 37)		
Intra-operative results								
Surgery time, minute	367.04 ± 120.00	350.05 ± 108.30	414.65 ± 144.84	< 0.001	341.70 ± 93.32	419.65 ± 143.39	< 0.001	0.887
CPBT, minute	169.66 ± 85.90	160.24 ± 80.39	199.53 ± 91.46	< 0.001	148.33 ± 67.35	207.70 ± 112.66	< 0.001	0.647
Aortic occlusion time, minute	93.82 ± 49.79	87.69 ± 44.49	116.66 ± 58.65	< 0.001	82.12 ± 40.00	106.38 ± 60.73	< 0.001	0.271
Minimum temperature, ℃	34.29 ± 1.95	34.33 ± 2.28	33.93 ± 1.98	0.172	34.60 ± 0.68	34.10 ± 1.63	0.018	0.287
Sevoflurane inhalation, (%)	385 (93.7)	195 (92.0)	76 (96.2)	0.206	77 (92.8)	37 (100.0)	0.093	0.478
Dexmedetomidine, (%)	266 (64.7)	145 (68.4)	47 (59.5)	0.154	53 (63.9)	21 (56.8)	0.460	0.405
Dopamine, (%)	56 (13.6)	24 (11.3)	12 (15.2)	0.373	15 (18.1)	5 (13.5)	0.536	0.248
Dobutamine, (%)	239 (58.2)	123 (58.0)	49 (62.0)	0.536	73 (88.0)	34 (91.9)	0.521	0.541
Norepinephrine, (%)	350 (85.2)	172 (81.1)	71 (89.9)	0.074	71 (85.5)	75 (90.4)	0.340	0.142
Adrenaline, (%)	66 (16.1)	33 (15.6)	13 (16.5)	0.853	12 (14.5)	8 (21.6)	0.331	0.829
Heart valve surgeries, (%)	237 (57.7)	118 (55.7)	47 (59.5)	0.405	47 (56.6)	25 (67.6)	0.259	0.538
CABG, (%)	57 (13.9)	39 (18.4)	2 (2.5)	0.001	13 (15.7)	3 (8.1)	0.261	0.840
VR + CABG, (%)	16 (3.9)	7 (3.3)	3 (3.8)	0.836	4 (4.8)	2 (5.4)	0.892	0.456
Vascular surgery, (%)	40 (9.7)	14 (6.6)	17 (21.5)	< 0.001	4 (4.8)	5 (13.5)	0.095	0.327
Congenital surgery, (%)	51 (12.4)	28 (13.2)	10 (12.7)	0.902	12 (14.5)	1 (2.7)	0.056	0.534
Other cardiac surgeries (%)	10 (2.4)	6 (2.8)	0 (0.0)	0.131	3 (3.6)	1 (2.7)	0.797	0.447
Fluid intake and output								
Blood loss, ml	566.67 ± 329.37	535.61 ± 301.60	702.53 ± 451.34	< 0.001	480.12 ± 185.60	648.65 ± 334.89	< 0.001	0.172
RBC, ml	375.47 ± 365.64	327.36 ± 328.53	525.13 ± 420.73	< 0.001	277.53 ± 330.27	551.35 ± 375.90	< 0.001	0.631
Plasma, ml	445.50 ± 430.60	388.68 ± 210.59	522.78 ± 302.00	< 0.001	525.30 ± 330.27	427.03 ± 245.68	0.479	0.135
Cryoprecipitate, U	0.73 ± 2.59	0.44 ± 2.12	1.04 ± 3.02	0.061	0.94 ± 2.90	1.22 ± 3.17	0.641	0.135
PLT, U	0.36 ± 0.56	0.32 ± 0.51	0.42 ± 0.63	0.195	0.30 ± 0.56	0.54 ± 0.65	0.041	0.679

*Abbreviations* CPBT, cardiopulmonary bypass time; CABG, Coronary Artery Bypass Grafting; RBC, red blood cell

### Characteristics selection and development of a nomogram

**Fig. 4:**
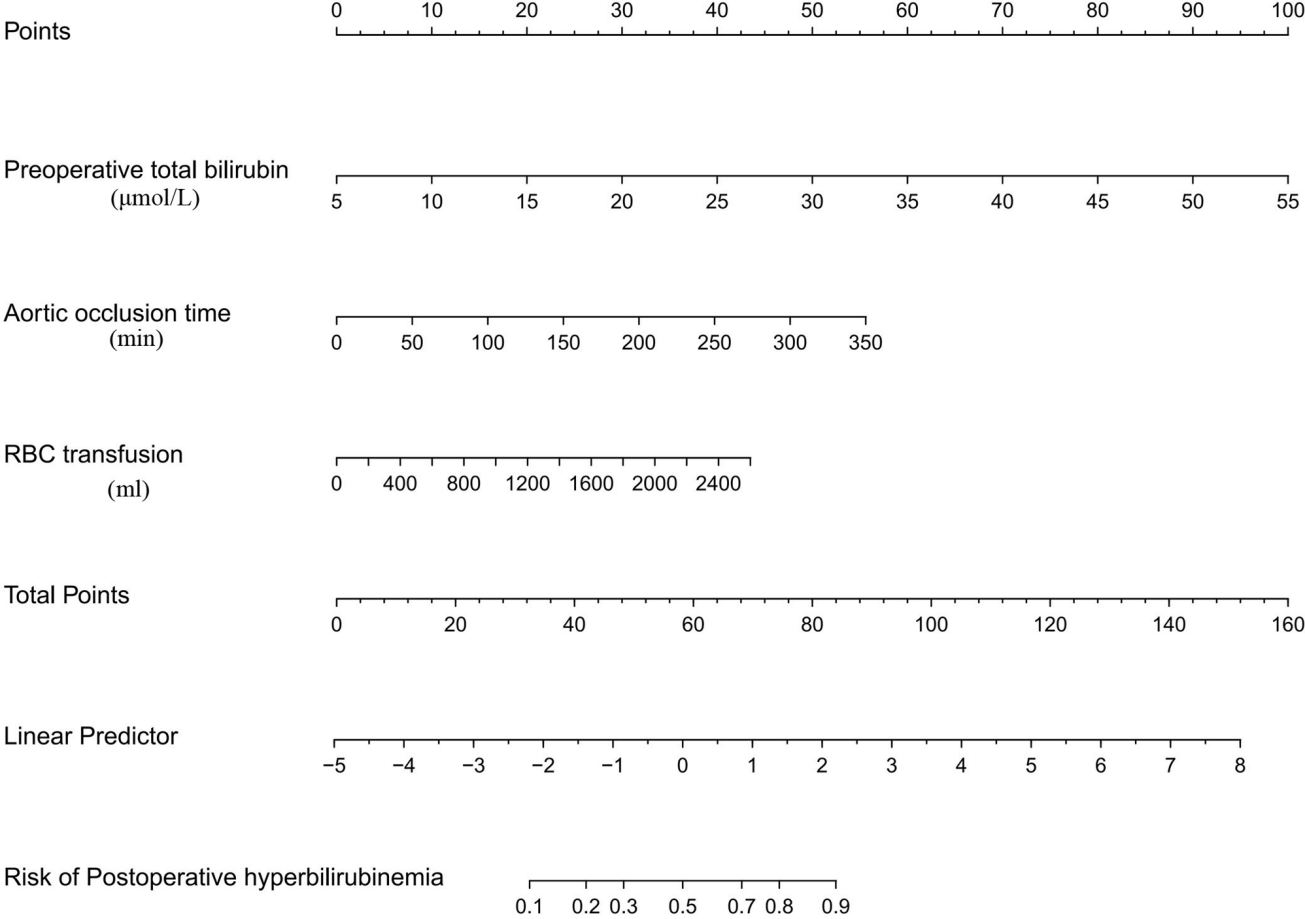
Nomogram of factors associated with the final model for diagnosing postoperative HB, including preoperative total bilirubin, aortic clamp time and RBC transfusion. First, a point was found for each variable on the uppermost rule; then, all scores were added together, and the total number of points was collected. Finally, the corresponding predicted probability of HB was found on the lowest rule

LASSO regression was conducted for 50 candidate variables, and 4 variables were selected (Table S2). The multivariate logistic analysis of these 4 variables revealed 3 risk factors to be included in the final model: preoperative total bilirubin (TBIL) (OR: 1.183; 95% *CI*: 1.130–1.245), aortic occlusion time (OR: 1.012; 95% CI: 1.005–1.020) and RBC transfusion (OR: 1.001; 95% CI: 1.001–1.002) (Table 3). A nomogram for predicting postoperative HB was constructed on the basis of the final model (Fig. 4).

**Table 3:** The multivariate logistic regression model of independent variables to HB

Parameters	OR	95% CI	*P*
TBIL	1.183	1.130,1.245	< 0.001
Aortic occlusion time	1.012	1.005,1.020	< 0.001
RBC transfusion	1.001	1.001,1.002	0.001
Vascular surgery	2.188	0.875,5.470	0.092

### Apparent performance and evaluation of the nomogram

**Fig. 5:**
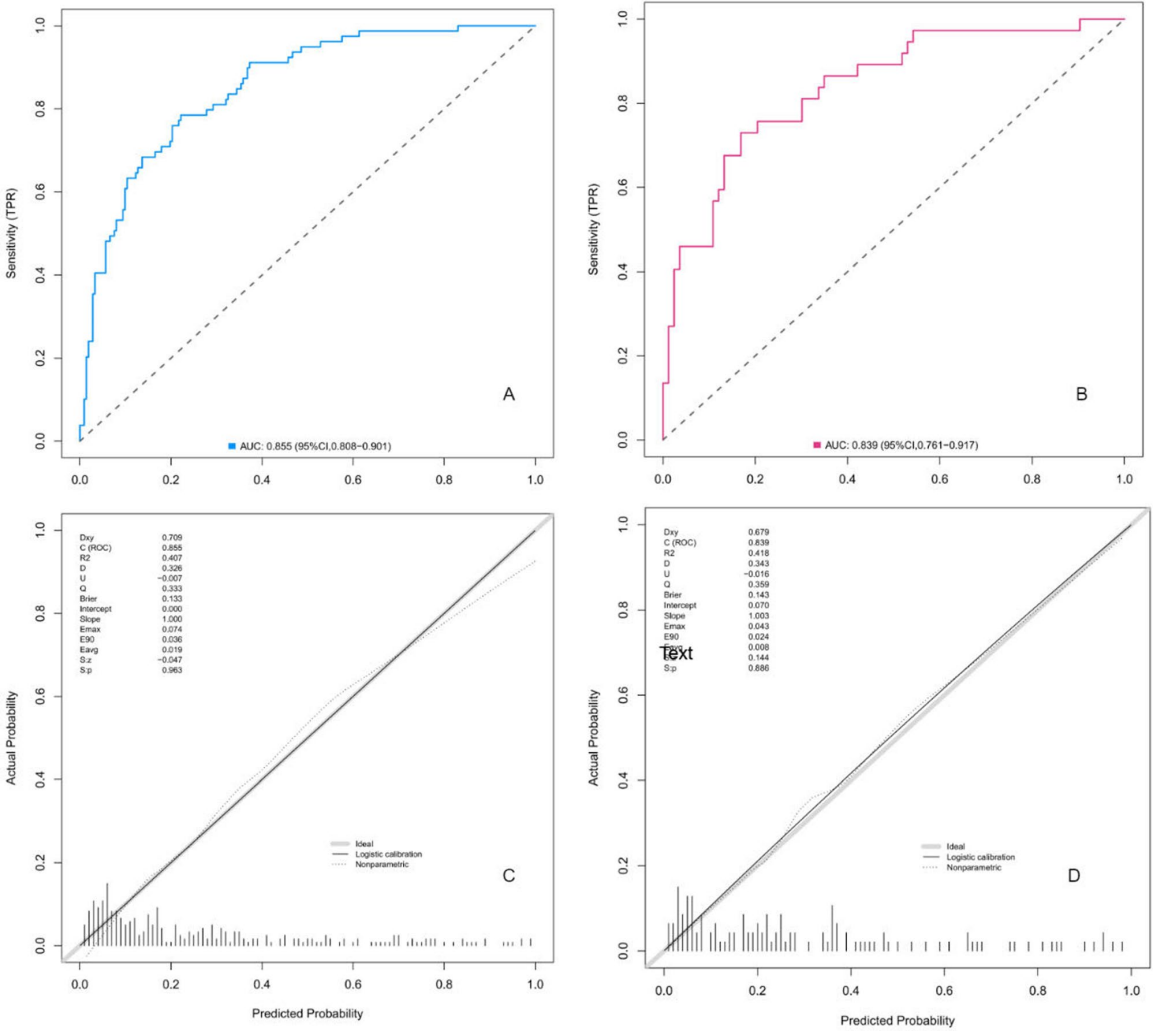
(**A**, **B**) ROC curves showing the model in predicting postoperative HB in the training and validation cohorts. (**C**, **D**) Calibration plot showing the degree of fit of the nomogram for the training and validation cohorts

The C-index of the prediction model in the training cohort was 0.854 (95% *CI* = 0.808–0.901), and Nagelkerke’s R2 was 0.95. The AUC of the prediction model for postoperative HB was 0.855, and the sensitivity, specificity, PPV, NPV and Youden’s index were 0.785, 0.778, 0.569, 0.901 and 0.563, respectively (Fig. 5A). In the validation cohort, the C-index of the prediction model in the validation cohort was 0.835 (95% *CI* = 0.761–0.917) after internal verification using 1000 bootstraps. The AUC of the prediction model for postoperative HB was 0.839, and the sensitivity, specificity, PPV, NPV and Youden’s index were 0.757, 0.747, 0.571, 0.873 and 0.504, respectively (Fig. 5B).

The calibration plot revealed a good degree of fit of the nomogram for both the training and validation cohorts (Fig. 5C and D). The Hosmer‒Lemeshow goodness-of-fit test (HL test) of multivariate analysis demonstrated excellent consistency between the predicted and observed values (training cohorts, χ^2^ = 7.804, df = 8, *P* = 0.453; validation cohorts, χ^2^ = 8.193, df = 8, *P* = 0.415).

### Restricted cubic splines between aortic occlusion time, RBC transfusion, preoperative TBIL and HB

**Fig. 6:**
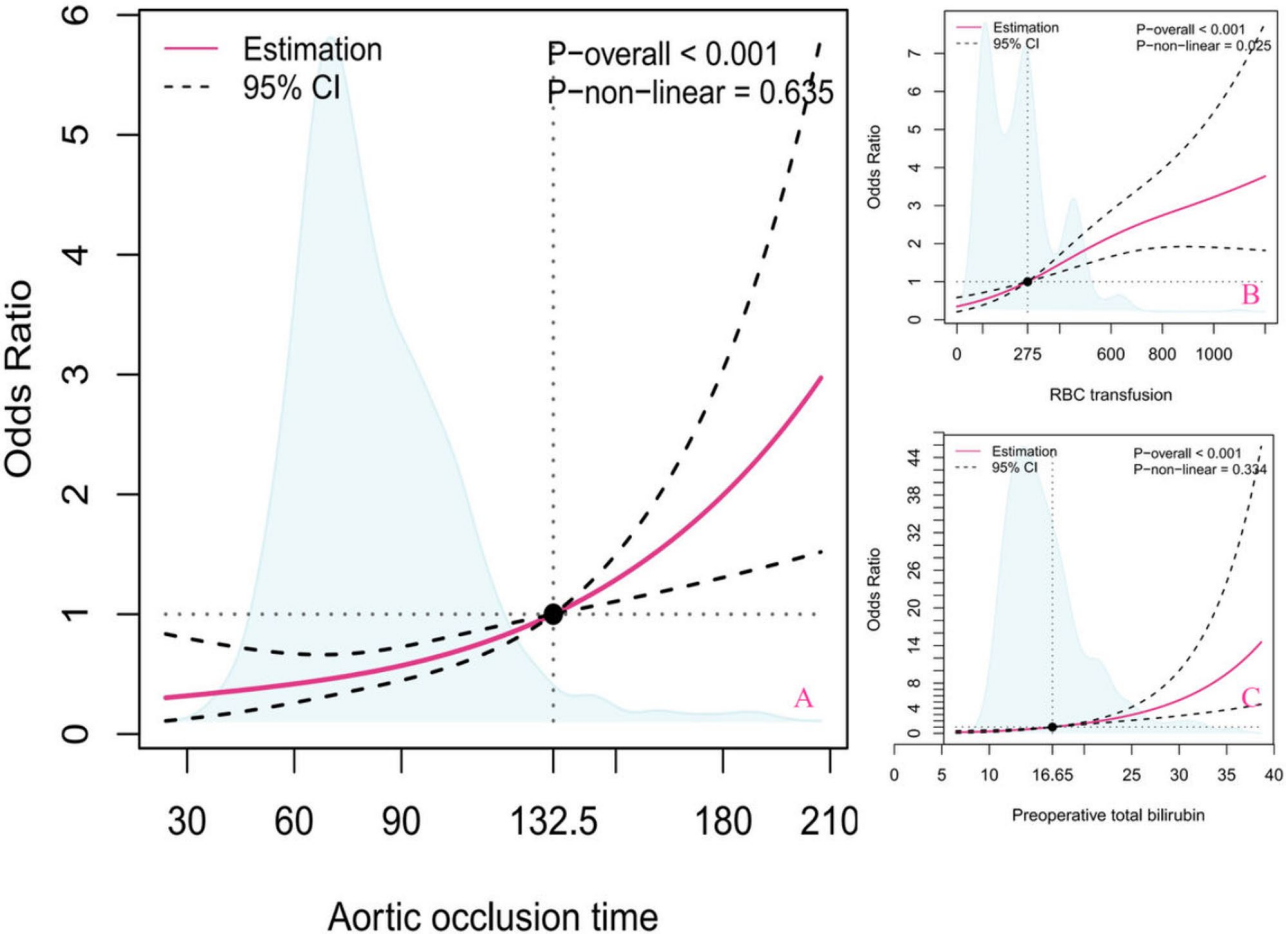
Restricted cubic splines between (**A**) aortic occlusion time, (**B**) RBC transfusion, and (**C**) preoperative total bilirubin level, with reference knots set at 132.5 mmHg, 275 ml and 16.65 µmol/L. The solid red lines are multivariable adjusted odds ratios, with dashed bold lines showing 95% confidence intervals derived from restricted cubic spline regressions. Reference lines for no association are indicated by the black dashed lines at a hazard ratio of 1.0

RCS confirmed a linear association between aortic occlusion time (*P*-overall < 0.001, *P*-nonlinear = 0.635; Fig. 6A), RBC transfusion (*P*-overall < 0.001, *P*-nonlinear = 0.025; Fig. 6B), preoperative TBIL (*P*-overall < 0.001, *P*-nonlinear = 0.334; Fig. 6C) and HB. The reference points for aortic occlusion time, RBC transfusion and preoperative TBIL were 132.5 min, 275 ml and 16.65 µmol/L, respectively. There was a linear relationship between the aortic occlusion time and preoperative TBIL and HB levels. The risk of HB increased linearly when the aortic occlusion time was above 132.5 min and the preoperative TBIL level was above 16.65 µmol/L.

### Patient postoperative outcomes

Patients with postoperative HB experienced longer intensive care unit (ICU) stays, ventilation times and postoperative hospital stays (PODs), as well as increased incidences of postoperative bleeding > 400 ml (1.7% vs. 0%, *P* < 0.05), CPR (3.4% vs. 0.7%, *P* < 0.05), 48 h-AKI (47.4% vs. 28.1%, *P* < 0.001), 7d-AKI (36.2% vs. 19.3%, *P* < 0.001), total AKI (49.1% vs. 29.8%, *P* < 0.001) and in-hospital death (12.9% vs. 3.7%, *P* < 0.05) (Table 4).

**Table 4:** Patient’s post-operative outcomes

Parameters	All (*n* = 411)	Non-HB (*n* = 295)	HB (*n* = 116)	*P*
Urine, ml	1545.83 ± 897.28	1496.47 ± 874.39	1671.34 ± 945.31	0.075
ICU stay, day	2.0 (1.0,3.0)	2.0 (1.0,2.5)	2.0 (1.0,4.0)	< 0.001
Ventilation time, h	12.0 (6.0,19.0)	9.0 (6.0,18.0)	16.5 (8.8,23.0)	< 0.001
Re-intubation, No. (%)	4 (1.0)	2 (0.7)	2 (1.7)	0.331
Bleeding > 400 ml, (%)	2 (0.5)	0 (0.0)	2 (1.7)	0.024
Secondary thoracotomy, (%)	1 (0.2)	1 (0.3)	0 (0.0)	0.530
CPR, (%)	6 (1.5)	2 (0.7)	4 (3.4)	0.035
IABP, (%)	19 (4.6)	13 (4.4)	6 (5.2)	0.739
ECMO, (%)	3 (0.7)	2 (0.7)	1 (0.9)	0.844
48 h-AKI, (%)	138 (33.6)	83 (28.1)	55 (47.4)	< 0.001
7d-AKI, (%)	99 (24.1)	57 (19.3)	42 (36.2)	< 0.001
AKI total, (%)	145 (35.3)	88 (29.8)	57 (49.1)	< 0.001
In-hospital stay, day	26.72 ± 10.92	26.17 ± 10.30	28.10 ± 12.29	0.107
POD, day	17.18 ± 9.26	16.55 ± 8.13	18.78 ± 11.54	0.027
In-hospital death	26 (6.3)	11 (3.7)	15 (12.9)	0.001

AKI, acute kidney injury; CPR, cardiopulmonary resuscitation; ECMO, extracorporeal membrane oxygenation

IABP, Intra-aortic balloon pump; ICU, intensive care unit; POD, Postoperative hospital stay

## Discussion

Understanding the risk factors for postoperative HB is crucial for preventing and improving surgical outcomes and patient prognosis. CPB-related liver damage can be linked to both proinflammatory syndromes, which include the production of hepatotoxic cytokines, and ischemic hepatitis and congestive hepatic stasis caused by hemodynamic abnormalities associated with surgery and extracorporeal circulation [14]. Several studies have identified various risk factors for HB after various cardiac surgeries [15–18]. In this study, a model that can predict postoperative HB after extracorporeal circulation surgery was developed and internally validated. Aortic clamp time was identified as an independent risk factor for high HB after extracorporeal circulation surgery, alongside blood transfusion volume and preoperative bilirubin. The risk of HB after extracorporeal circulation significantly increased when the aortic clamp time was greater than 133 min.

The incidence of HB in the present study was 28.2%, which closely aligns with findings from other studies [2, 6, 8, 15, 19–21]. Moreover, most cases of HB peaked on the first day after surgery, which was observed in 77.2% of patients. Similarly, the incidence of HB was greater in valve surgeries than in CABG and other cardiac surgeries. Major vascular surgery is reviewed as a surgically complex procedure that involves several steps, potentially leading to a higher transfusion requirement and subsequent HB than other surgeries with the same aortic block time. Additionally, increased bleeding and greater intraoperative suction pressure in large vessel surgeries could contribute to higher red blood cell destruction.

Although most studies revealed a difference in aortic clamp duration between the postoperative HB and non-HB groups, not all studies concluded that the aortic clamp time was a risk factor for postoperative HB [5, 6, 8, 22]. Our study corroborates the findings of Pranav Sharma that aortic clamp duration is an independent risk factor for postoperative HB following extracorporeal circulation. This finding was also supported by a meta-analysis [7]. Most importantly, our research goes a step further by identifying a threshold of 133 min as a critical point for an increased risk of HB. This finding could explain the discrepancy observed in previous studies regarding the determination of aortic clamp time as a significant risk factor [8, 22]. The reason for this discrepancy might be that most of the past studies had aortic clamp times shorter than the critical value of 133 min, which consequently allowed the impact of the aortic clamp time on the risk of HB to be underestimated or not detected. The RCS curve analysis further revealed that the risk of HB significantly increases when the aortic clamp period exceeds 133 min, which might account for the increased risk of HB in multivalve surgery and aortic coarctation.

Chen et al. and An et al. identified intraoperative blood transfusion volume as an important risk factor for HB after cardiopulmonary bypass [7, 8]. In our study, the risk of postoperative hyperbilirubinemia increased when the transfusion volume exceeded 275 ml. Despite the relatively small volume (slightly above 1 unit of red blood cells), the possibility of HB due to destruction of red blood cells in the stocked blood itself is low. Instead, we hypothesized that HB could be caused by the loss and destruction of red blood cells during intraoperative CPB due to contact with tubing [8, 23, 24].

Elevated preoperative total bilirubin was also consistently reported as a risk factor in previous studies [8, 19]. Our study supports this finding, in which the RCS analysis revealed a significant risk of HB when preoperative TBIL levels exceeded 16.65 mmol/L. This value is close to the upper limit of normal (17.1 mmol/L), indicating that even mild preoperative TB elevation is a risk factor for postoperative HB (OR = 1.183, 95% CI 1.130–1.245; *P* = 0.001). This evidence supports the notion that reduced liver reserve capacity and susceptibility to compensation loss could lead to postoperative HB3 [15].

The strength of our study lies in the development of a predictive model for postoperative HB in cardiac surgery patients via LASSO regression with a reduced number of parameters, enhancing its applicability. Additionally, by refining the aortic clamp time into a risk factor at 133 min, surgeons can be provided with better time references for surgical planning and implementation. However, our study had several limitations. First, this study lacks external validation, and its validity needs to be further confirmed. As a retrospective study, further analysis of the involved factors using a randomized control trial is warranted. Second, due to missing data, we were unable to determine whether the elevation in total bilirubin originated from unconjugated bilirubin (UCB) or conjugated bilirubin (CB). This needs to be addressed in a prospective or follow-up study. Third, preoperative right atrial pressure data were not available, as cardiac catheterization was not routinely performed. Fourth, blood loss significantly increased in both groups with increased preoperative bilirubin, which makes RBC transfusions probable. Including the amount of RBC volume given as a variable in a prediction model might be confounded by the fact that patients with increased HB will bleed more. Fifth, the sensitivity and specificity are well balanced, and the model is reliable when the model predicts a negative outcome. The moderate positive predictive value suggests some false positive inclusions. This may lead to unnecessary interventions or follow-up. If the cost or risk of false positives is high, this might be a limitation.

## Conclusion

In conclusion, our study emphasized that the aortic clamp time, rather than the CPB time, is a critical factor associated with postoperative HB during cardiac surgery. Elevated preoperative bilirubin, aortic clamp time, and intraoperative RBC transfusion volume serve as valuable predictors of postoperative HB.

## Electronic supplementary material

Below is the link to the electronic supplementary material.

Supplementary Material 1: Figure S1 Plots for LASSO regression coefficients

Supplementary Material 2: Figure S2 The cross-validation plot for the penalty term (λ)

Supplementary Material 3

Supplementary Material 4

Supplementary Material 5

## Data Availability

Data is provided within the manuscript or supplementary information files.

## Abbreviations

AUC: Area under the curve
CABG: Coronary artery bypass grafting
CPB: Cardiopulmonary bypass
DCA: Decision curve analysis
HB: Hyperbilirubinemia
HTK: Histidine-Tryptophan-Ketoglutarate
ICU: Intensive care unit
IQR: Interquartile range
LASSO: Least absolute shrinkage and selection operator
MELD: Model for the prognosis of end-stage liver disease
NPV: Negative predictive value
PLT: Platelet
PPV: Positive predictive value
OR: Odds ratio
RBC: Red blood cell
RCS: Restricted cubic splines
ROC: Receiver operating characteristic
SD: Standard deviation
TBIL: Total bilirubin
