# Supplementary figures and images for: Risk assessment of hyperbilirubinemia using a three-factor model after cardiac surgery
Source: BMC Surg. 2025 Feb 13;25:63. doi: 10.1186/s12893-024-02731-6 (PMC11823160; doi:10.1186/s12893-024-02731-6)

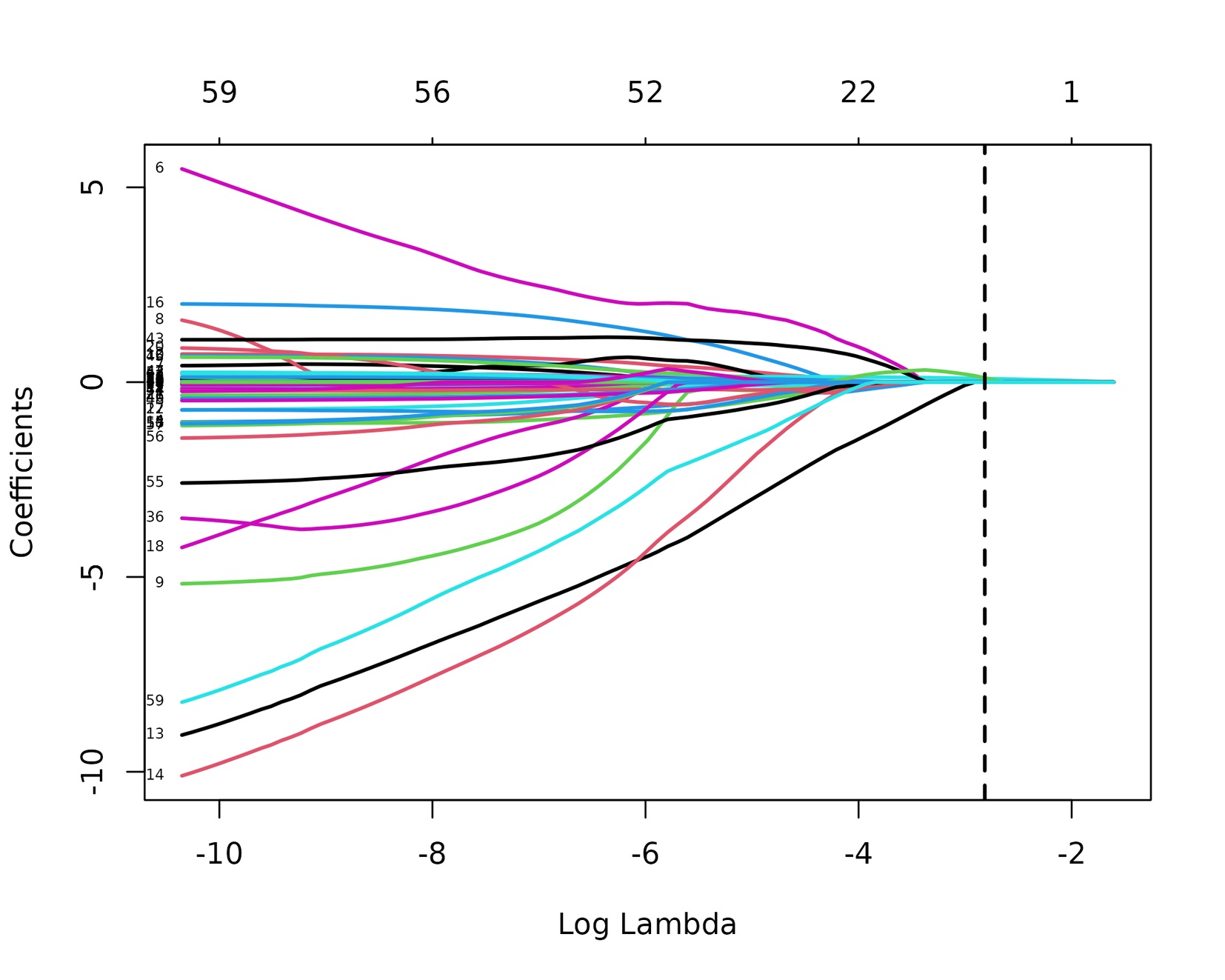

Supplement: Supplementary file 1 — Supplementary Material 1: Figure S1 Plots for LASSO regression coefficients [file 12893_2024_2731_MOESM1_ESM.jpg]

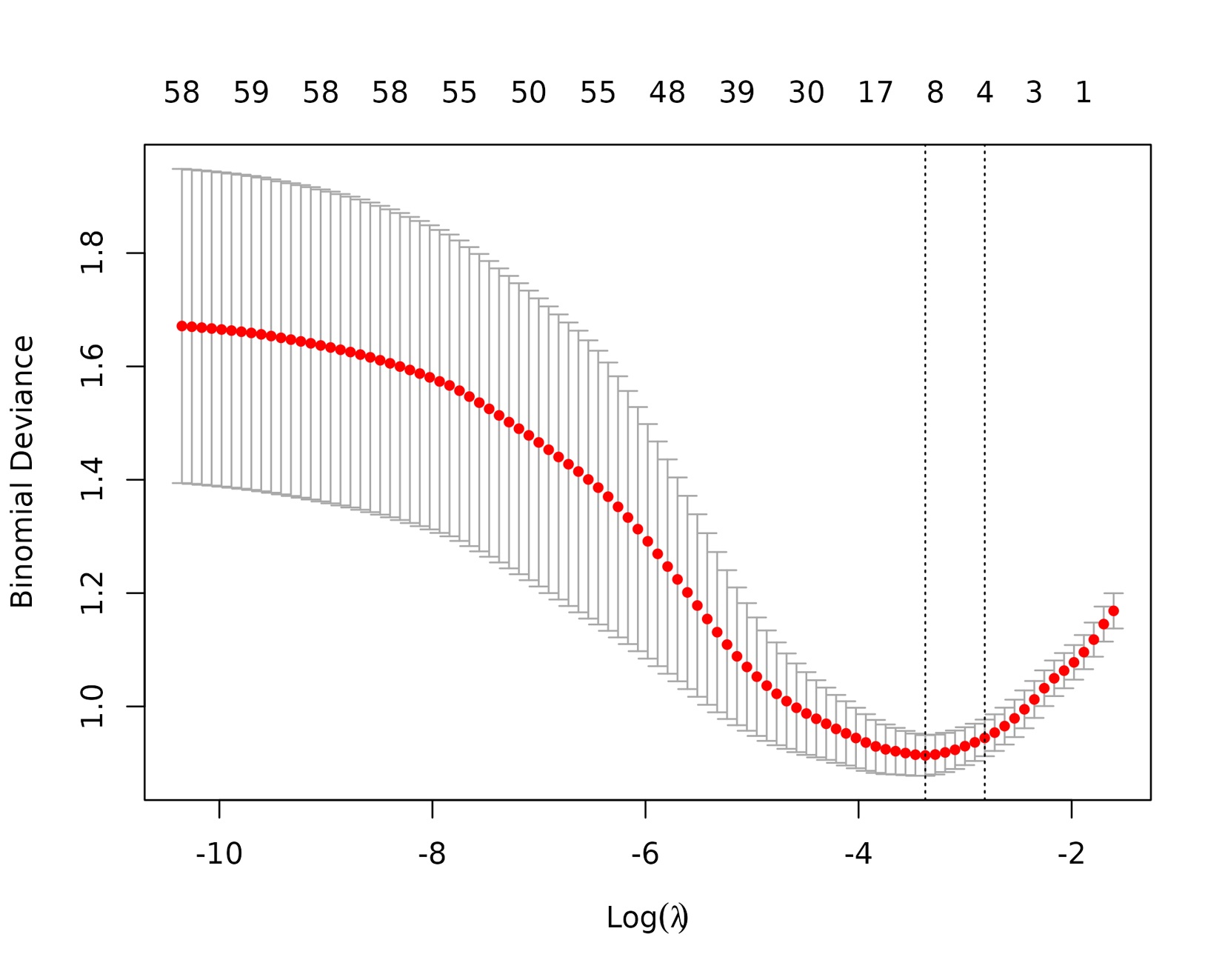

Supplement: Supplementary file 2 — Supplementary Material 2: Figure S2 The cross-validation plot for the penalty term (λ) [file 12893_2024_2731_MOESM2_ESM.jpg]
