# Supplementary material for: Risk assessment of hyperbilirubinemia using a three-factor model after cardiac surgery
Source: BMC Surg. 2025 Feb 13;25:63. doi: 10.1186/s12893-024-02731-6 (PMC11823160; doi:10.1186/s12893-024-02731-6)
Supplement: Supplementary file 3 — Supplementary Material 3 [file 12893_2024_2731_MOESM3_ESM.docx]

**Table S1** **The incidence and comparison of HB after different cardiac surgeries**

| Parameters | All (*n*=411) | Non-HB (*n*=295) | HB (*n*=116) | Incidence rate（%） | *P* |
| --- | --- | --- | --- | --- | --- |
| Heart valve surgery (%) | 237 (57.7) | 165 (55.9) | 72 (62.1) | 30.7^a^ | .257 |
| CABG (%) | 57 (13.9) | 52 (17.6) | 5 (4.3) | 11.9^b^ | <.001 |
| VR+CABG (%) | 16 (3.9) | 11 (3.7) | 5 (4.3) | 35.0^a,b,c^ | .784 |
| Vascular surgery (%) | 40 (9.7) | 18 (6.1) | 22 (19.0) | 53.5^c^ | <.001 |
| Congenital (%) | 51 (12.4) | 40 (13.6) | 11 (9.5) | 21.6^a,b^ | .259 |
| Other cardiac surgeries (%) | 10 (2.4) | 9 (3.1) | 1 (0.9) | 10.0^a,b,c^ | .195 |

Abbreviations: CABG,coronary artery bypass surgery;VR,Valve replacement.

Heart valve surgery：CABG<0.05；Heart valve surgery：Vascular surgery<0.05；

CABG：Vascular surgery<0.05；

Vascular surgery：Congenital<0.05；

Congenital ：Other cardiac surgeries<0.05.
