# Supplementary material for: Risk assessment of hyperbilirubinemia using a three-factor model after cardiac surgery
Source: BMC Surg. 2025 Feb 13;25:63. doi: 10.1186/s12893-024-02731-6 (PMC11823160; doi:10.1186/s12893-024-02731-6)
Supplement: Supplementary file 4 — Supplementary Material 4 [file 12893_2024_2731_MOESM4_ESM.docx]

**Table S2 Lasso test (Lambda.1 se. Coef）**

| **Parameters** | **coef** |
| --- | --- |
| (Intercept) | -3.075752991 |
| TBIL | 0.091576201 |
| Aortic occlusion time | 0.004882218 |
| RBC transfusion | 0.000500611 |
| Vascular surgery | -0.10876418 |

Lambda.1 se: 0.065217391304347.
