# Supplementary material for: Risk assessment of hyperbilirubinemia using a three-factor model after cardiac surgery
Source: BMC Surg. 2025 Feb 13;25:63. doi: 10.1186/s12893-024-02731-6 (PMC11823160; doi:10.1186/s12893-024-02731-6)
Supplement: Supplementary file 5 — Supplementary Material 5 [file 12893_2024_2731_MOESM5_ESM.docx]

**Table S3 Variance Inflation Factor and Tolerance**

| Term | VIF | VIF_CI_low | VIF_CI_high | SE_factor | Tolerance | Tolerance_CI_low | Tolerance_CI_high |
| --- | --- | --- | --- | --- | --- | --- | --- |
| TBIL | 1.156113 | 1.06079 | 1.401377 | 1.075227 | 0.8649675 | 0.7135840 | 0.9427567 |
| Aortic occlusion time | 1.139529 | 1.049820 | 1.390773 | 1.067487 | 0.8775555 | 0.7190246 | 0.9525442 |
| RBC transfusion | 1.069680 | 1.011100 | 1.437418 | 1.034253 | 0.9348594 | 0.6956917 | 0.9890221 |
